# Myopic choroidal neovascularization with neovascular signal around perforating scleral vessel prone to recur after anti-VEGF therapy

**DOI:** 10.1186/s40662-024-00374-5

**Published:** 2024-02-07

**Authors:** Xiangjun She, Wangjing Yao, Gongyu Huang, Zhi Liang, Jin Xie, Jiwei Tao, Sulan Wu, Jianbo Mao, Yiqi Chen, Yun Zhang, Lijun Shen

**Affiliations:** 1 National Clinical Research Center for Ocular Diseases, Eye Hospital, Wenzhou Medical University, Wenzhou, 325027 China; 2 Center for Rehabilitation Medicine, Department of Ophthalmology, Zhejiang Provincial People’s Hospital (Affiliated People’s Hospital, Hangzhou Medical College), 158, Shangtang Road, Hangzhou, 310014 Zhejiang China

**Keywords:** Perforating scleral vessel, Choroidal neovascularization, Pathological myopia, Anti-VEGF therapy, Optical coherence tomography angiography

## Abstract

**Background:**

To compare the recurrence of myopic choroidal neovascularization (mCNV) based on the neovascular signal of mCNV around the perforating scleral vessel (PSV).

**Methods:**

A consecutive series of naïve patients with mCNV accepted anti-VEGF therapy with a minimum 12-month follow-up period. The neovascular signal relationship between PSV and mCNV were classified into the presence of neovascular signal of CNV around PSV or not. The recurrence of mCNV, best-corrected visual acuity (BCVA), hyperreflective foci height, CNV area and CNV flow area were analyzed between two groups.

**Results:**

Neovascular signal of CNV around PSV was detected in 20 eyes (39.2%). The one-year recurrence rate in the group with neovascular signal of CNV around PSV was significantly higher than that in the group without neovascular signal of CNV around PSV (*P* = 0.045). The recurrence time in the group with neovascular signal around PSV was shorter than that in the group without neovascular signal around PSV (*P* = 0.030). Cox proportional hazard model showed that the presence of neovascular signal of CNV around PSV [hazard ratio (HR): 2.904] and subfoveal choroidal thickness ≤ 50 μm (HR: 0.368) were risk factors for recurrence of mCNV. In the group with neovascular signal around PSV, the BCVA was worse (*P* = 0.024) and the CNV flow area was more unstable (*P* = 0.027) after therapy.

**Conclusions:**

PSV was commonly detected in patients with mCNV. The presence of neovascular signal of CNV around PSV was prone to recur with a shorter time in mCNV patients.

**Supplementary Information:**

The online version contains supplementary material available at 10.1186/s40662-024-00374-5.

## Background

Myopic choroidal neovascularization (mCNV) is one of the complications of pathologic myopia, commonly resulting in severe visual impairment [1]. The natural course of long-term visual outcomes in patients with mCNV is unfavorable. Most patients with mCNV experience a decline in visual acuity (VA) to 20/200 or below in 5 to 10 years following the onset with the development of CNV-related macular atrophy [2]. Currently, intravitreal anti-vascular endothelial growth factor (anti-VEGF) injection is the first-line treatment for mCNV [3]. Recent clinical trials have shown that anti-VEGF therapy is safe and can considerably increase visual acuity in patients with mCNV [1, 4]. Rebleeding of mCNV was reported in 15%–20% patients at a follow-up of 10 years without treatment. However, even after treatment, 29.4%–46.1% patients with mCNV frequently experience recurrence [5–7]. The thinner choroidal thickness and larger CNV size were considered as risk factors for recurrence after anti-VEGF treatment [8, 9].

The latest study proposed that mCNV was closely related to perforating scleral vessels (PSV). PSV was detected in 80% of mCNV [10]. Ohno-Matsui’s study reported that CNV in pathological myopia had a connection with short posterior ciliary arteries [11]. Querques et al. speculated that the scleral dilatation at the location of the perforating vessels may play a role in the formation of lacquer crack which is a risk factor for CNV [12]. Further study was reported that patients with mCNV and PSV need more injections and have a higher recurrence rate than mCNV without PSV [13]. The role of PSV in mCNV was not clear.

In our earlier study, we found that when PSV was adjacent to mCNV, the VA outcome was poorer [14]. It was unclear whether the location of flow signal of PSV with mCNV were related to recurrence. Therefore, in this study, we analyzed the neovascular signal relationship between PSV and mCNV by optical coherence tomography angiography (OCTA) and evaluated its clinical significance with recurrence of mCNV after therapy.

## Methods

This retrospective study was performed at the Eye Hospital of Wenzhou Medical University in China. The study was approved by the Ethics Committee of Eye Hospital of Wenzhou Medical University, Zhejiang Province, China (H2022-010-K-10-01), and followed the tenets of the Declaration of Helsinki. The medical records of patients who visited the Eye Hospital of Wenzhou Medical University between January 2017 and December 2021 were reviewed. The need for participation consent was waived due to the retrospective nature of this study. Written informed consent for all performed surgical procedures was obtained from each patient prior to scheduled surgery.

The inclusion criteria were: (1) Meet the criteria of pathological myopia: the refractive error (spherical equivalent) is at least − 6.00 diopter (D) or the axial length is more than 26.00 mm, with typical sclera, choroid and retina degeneration; (2) Existence of active CNV: comprehensive interpretation of fundus photography, optical coherence tomography (OCT), OCTA and fluorescein angiography (FA); (3) The first anti-VEGF therapy was performed in this hospital; and (4) Follow-up time ≥ 12 months. The exclusion criteria were: (1) At the first diagnosis, CNV was in the stage of scar or atrophy; (2) CNV was secondary to other diseases, such as punctate inner choroidopathy, multifocal choroiditis, age-related macular degeneration; (3) During the follow-up period, the eyes that underwent intraocular surgery in addition to anti-VEGF therapy; (4) Combining with severe posterior segment complications, such as retinal detachment or macular hole; (5) Previous history of anti-VEGF therapy in other institutions; and (6) CNV outside a 3 × 3 mm^2^ on OCTA image, or CNV too large to fit within the 3 × 3 mm^2^ area.

The age, sex, best-corrected VA (BCVA) and refractive error were collected from the medical records of the initial examination. The dilated fundus examination that included indirect ophthalmoscopy, color fundus photography (TRC-50DX, Topcon Corporation, Tokyo, Japan) or confocal scanning laser ophthalmoscopy (cSLO; Optos Daytona, Optos, England), structural spectral-domain OCT (SD-OCT) (Spectralis SD-OCT; Heidelberg Engineering, Germany), OCTA (Angio OCT; Optovue, Fremont, America) and fundus fluorescein angiography (FFA)/indocyanine green angiography (ICGA), (HRA Spectralis, Heidelberg, Germany) were performed. BCVA, OCTA and OCT were recorded before treatment, 1 month, 3 months, 6 months and 12 months after therapy. Hyperreflective area height, central foveal thickness (CFT), subfoveal choroidal thickness (SFCT), CNV area and CNV flow area were measured before and after therapy.

### The classification of PSV based on B-scan and OCTA

PSV was defined as: (1) hyporeflective appearance presenting as linear or wavy morphology extension from the sclera through the choroid toward the retina in OCT B-scan images; and (2) the corresponding projection on en-face structure image presented as a black low-reflection lumen-like structure [15].

The location of neovascular signal between PSV and CNV were mainly analyzed through images of 512 consecutive horizontal and vertical B-scans on the 3 × 3 mm^2^ using OCTA (Angio OCT; Optovue, Fremont, America), by RTVue XR Avanti OCT system (Optovue Inc., Fremont, CA, USA), and the manufacturer’s AngioVue software (version number 2018.1.0.43) to observe the images. This version uses 3D projection artifact elimination technology to eliminate projection artifacts in en-face OCTA images and OCTA B-scans images, making image recognition more accurate [16]. All images were analyzed for segmentation errors and projection artifacts firstly and adjustments were made if necessary.

**Fig. 1:**
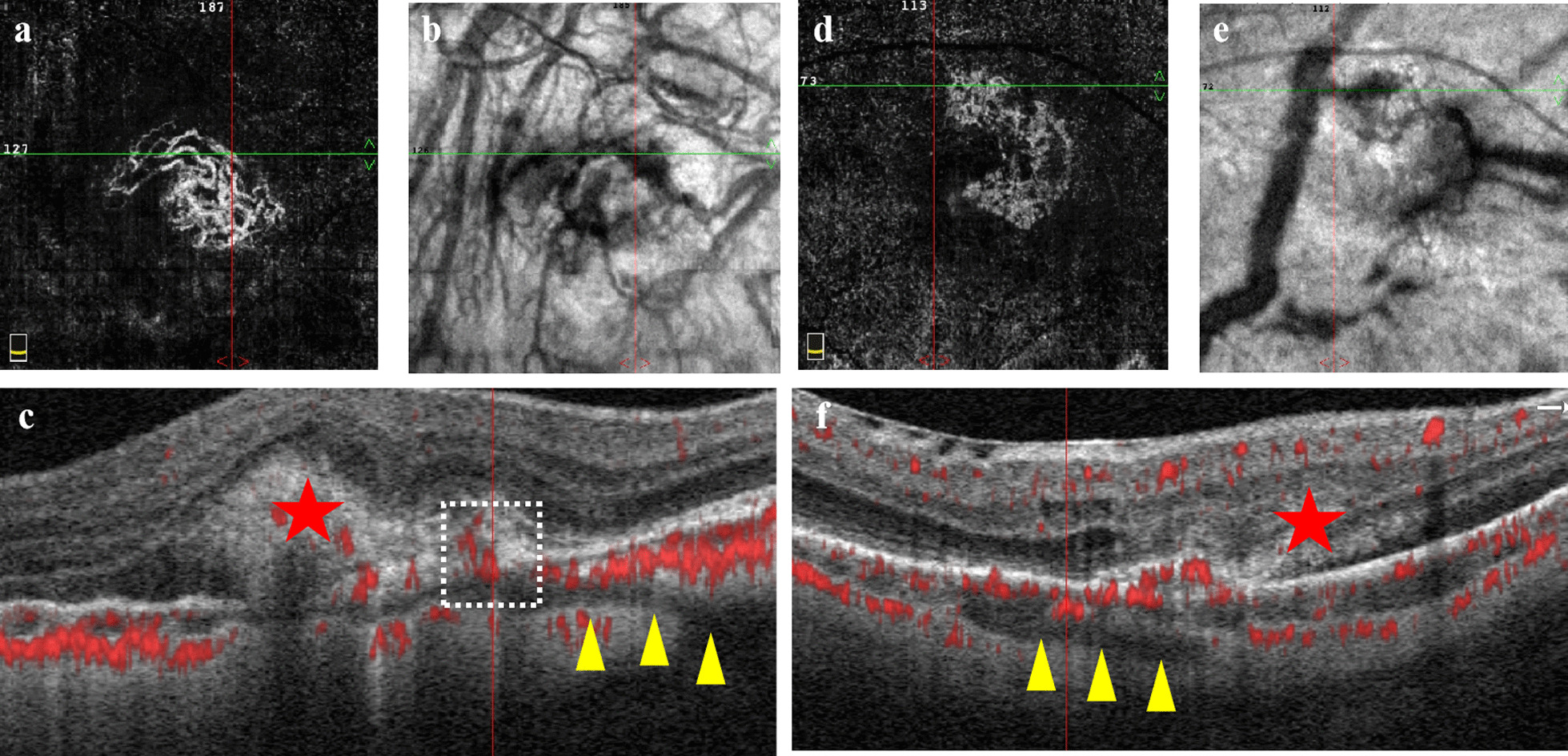
The morphology of perforating scleral vessel (PSV) based on the neovascularization signal by optical coherence tomography angiography (OCTA). **a–c** Presence of neovascular signal around PSV. **a** En-face OCTA image shows the shape of choroidal neovascularization (CNV) in the outer retinal layer, and the crossed lines indicate the location of PSV, which can correspond to the hyperreflection signal of CNV. **b** The corresponding en-face structure image of **a**. **c** The horizontal cross-sectional OCTA B-scan image. The yellow arrowhead indicates PSV, showing a low reflection linear hyporeflection structure to the choroid inclined from the sclera region, the red star indicates the hyperreflection exudation focus of mCNV, and the white box shows the blood flow signal (red flow signal) of neovascularization at the interface between the hyporeflection cavity and the hyperreflection focus. **d–f** Absence of neovascular signal around PSV. **d** En-face OCTA image shows the shape of CNV in the outer retinal layer, and the crossed lines indicate the location of PSV, which can not correspond to the hyperreflection signal of CNV. **e** The corresponding en-face structure image of **d**. **f** The horizontal cross-sectional OCTA B-scan image. The yellow arrowhead indicates PSV, showing a low reflection linear hyporeflection structure to the choroid inclined from the sclera region, the red star indicates the hyperreflection exudation focus of mCNV, and the flow signal of CNV does not intersect with PSV

**Fig. 2:**
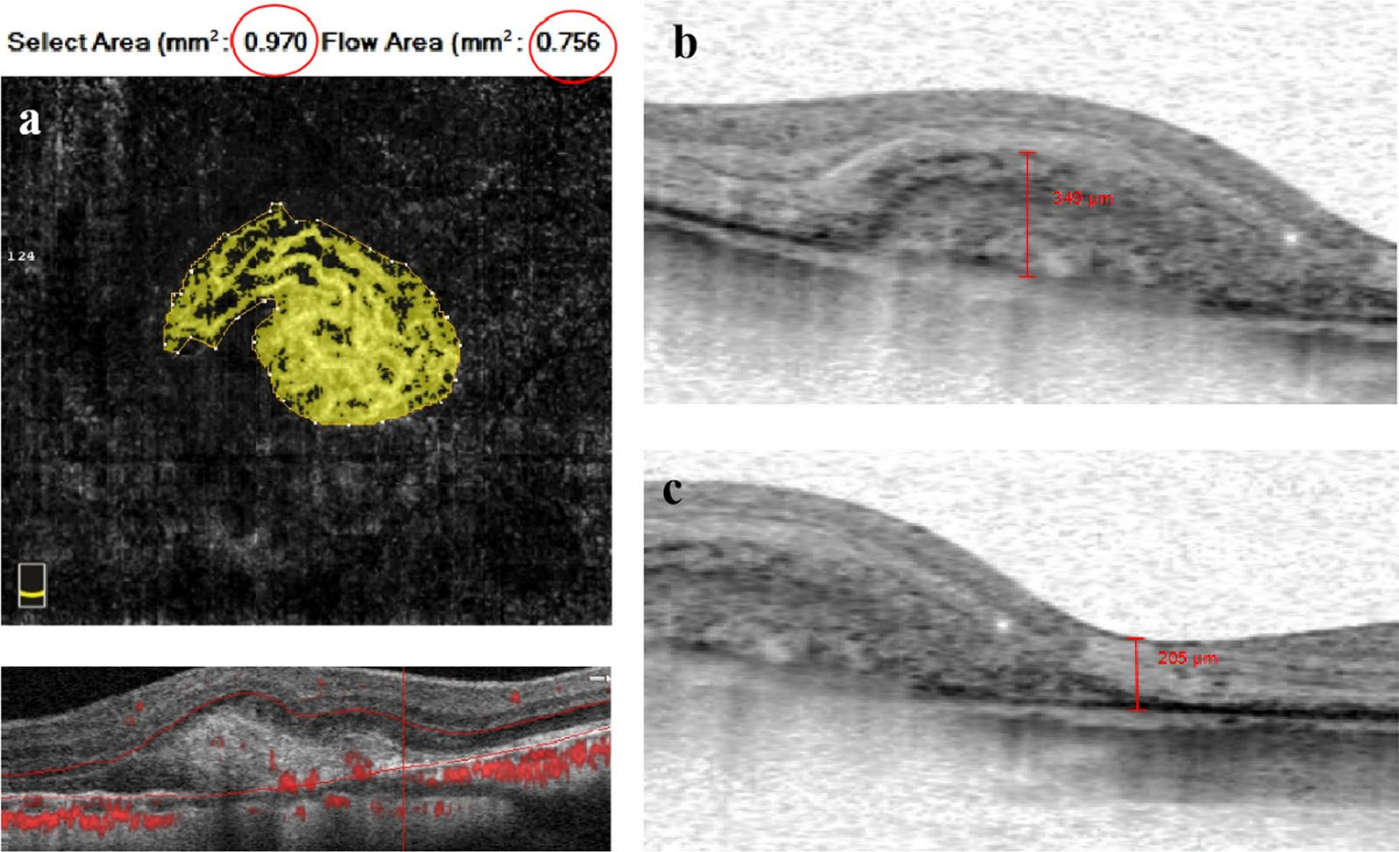
Schematic diagram of choroidal neovascularization (CNV) area, hyperreflective area height, central foveal thickness (CFT) measurement. **a** The CNV contour is selected manually, the CNV area (contour area) and CNV flow area are calculated automatically by using the built-in software (red circle). **b** The hyperreflective area height is the vertical distance between the highest point of hyperreflective area and Bruch’s membrane. **c** CFT is the vertical distance between the retinal pigment epithelium and the outer edge of the internal limiting membrane of the fovea

The location of flow signal from mCNV around PSV were evaluated by en-face OCTA images and B-scan OCTA images. The location of PSV under the CNV were observed through both horizontal and vertical cross-sectional OCTA B-scan. The neovascular signal of mCNV with PSV was divided into two groups: (1) Presence of neovascular signal around PSV—neovascular signal (red blood flow signal) can be observed adjacent to PSV which seemed like the neovascularization existed at the junction of PSV (linear low reflection structure) and CNV (high reflection exudate focus) (Fig. 1a–c); (2) There was no neovascular signal around PSV—no blood flow signal was observed at the junction of PSV and CNV (Fig. 1d–f). Two independent retina specialists classified all images on different days using the same monitor (the monitor resolution: 1920 × 1080 px) and any discrepancies were resolved with further discussion, so all signal types matched.

### The morphology of mCNV analyzed by OCTA

The morphology of mCNV was analyzed by en-face OCTA according to previous research. CNV patterns were classified, including “Medusa” pattern, “Seafan” pattern, “indistinct network” pattern and “pruned vascular tree” pattern, based on the presence of thin branches, circular peripheral anastomosis, feeder vessel, filamentous flow and dark halo [17].

The area of mCNV were measured on en-face OCTA images between Bruch's membrane and the outer plexiform layer which was automatically segmented by the AngioVue software. If the automatic stratification was not accurate due to pathological changes, it would be adjusted manually. The CNV area (contour area) and CNV blood flow area selected by the frame can be calculated automatically by using the built-in software along the CNV edge contour (Fig. 2a). The measurement is carried out independently by two doctors, and if the difference in data is more than 10%, it will be measured again [18].

The location of CNV was divided into subfoveal CNV and parafoveal CNV, according to whether CNV is less than 1 mm in diameter of the macular fovea.

### Therapy plan with anti-VEGF treatment

All patients were naïve without any treatment before. A 1 + pro-re-nata (PRN) regimen was used to conduct the study using 0.5 mg of ranibizumab or conbercept. When the mCNV activity was completely quiet at the next check-up, no further injections were given unless a recurrence was observed. If the mCNV activity remained, further injections were given monthly until the mCNV stabilized. The graders of the images with mCNV were masked to the patients’ treatment and clinical course.

### Definition of recurrence in mCNV

CNV recurrence was defined as a new development of dye leakage observed by FA, or a new or increased subretinal exudation-like serous retinal detachment observed in OCT images at least three months after the original fluid was completely resolved. Reactivation, enlargement of the original CNV, and a new CNV that developed away from the original CNV were the three categories into which the recurrences were categorized which was defined by Xie et al. Reactivation was shown by the increase of re-exudate on the B-scan, but there was no significant increase in CNV area. Enlargement of the original CNV was shown that not only the exudation increases, but also the area of CNV is larger than that of the original CNV by en-face OCTA. A new CNV was shown by the emergence of a new CNV away from the original CNV [19]. While observing the recurrence, B scan and OCTA are used to determine whether CNV has PSV at the site of recurrence, including their location and neovascular signal relationship.

### The measurement of hyperreflective area height, CFT, SFCT

The built-in caliper in the SD-OCT was used to measure these parameters. Hyperreflective area is defined as the area whose reflection is equal to or greater than the retinal pigment epithelium (RPE) layer. The height of hyperreflective area is defined as the vertical distance between the highest point of the hyperreflective area and Bruch’s membrane (Fig. 2b). CFT is defined as the vertical distance between the retinal pigment epithelium of the macular fovea and the outer edge of the internal limiting membrane (Fig. 2c). SFCT is defined as the vertical distance between the outer edge of the retinal pigment epithelium and the choroidoscleral interface. Each eye was measured three times by the same doctor and averaged.

### Definition of dilated choroidal vein (DCV)

The presence of DCV in the macular area was determined by ICGA and OCTA. DCV showed that the diameter of the great choroidal vein in the ICGA image was greater than or equal to two times the diameter of the adjacent vein [20].

### Statistical analysis

Statistical analysis was performed with a commercial program (SPSS for Windows; version 26.0.0; SPSS Inc., Chicago, IL). Snellen visual acuity was converted to the logarithm of the minimal angle of resolution (logMAR) for statistical purposes. The Kolmogorov–Smirnov test was used to determine the normality of all continuous variables. If the data was normally distributed, it was expressed as mean ± standard deviation; otherwise, it was expressed as the median (interquartile range). The independent *t*-test or Mann–Whitney test were used to compare continuous variables, and the Pearson’s χ^2^ test was used to compare categorical variables.

The Kaplan–Meier survival analysis and Cox proportional hazard model were performed for the first recurrence. Repeated measurement analysis of variance (RM-ANOVA) was used to compare the BCVA, hyperreflective area height, CFT, SFCT, CNV area and CNV flow area at each time point. *P* < 0.05 was considered statistically significant.

## Results

### Clinical study data

The medical records of 202 patients (207 eyes) with mCNV who received anti-VEGF therapy for the first time in the hospital were reviewed retrospectively. One hundred and fifty-six eyes were excluded: 72 had a follow-up period of less than 12 months, 66 had missing or incomplete critical follow-up data, 8 had image quality less than three leading to blurred angiography, 4 had other internal surgery, 3 had retinal detachment during the follow-up period and 3 had suspicious age-related macular degeneration. Further research was conducted on 51 eyes from 46 patients. Among all the cases, 3 had hypertension, no diabetes, 14 had cataract surgery before the observation period and all the ethnic groups were Han people.

Table 1 displays the demographic information of the 51 eyes. The average age was 53.88 ± 14.01 years old, with a refractive error of − 12.46 ± 3.12 D. The median baseline BCVA was 0.70 (0.40–1.30) logMAR, and the follow-up duration was 27 (20–41) months. 33 (64.7%) eyes had been diagnosed with subfoveal CNV while 18 (35.3%) with parafoveal CNV. The percentage of “Medusa”, “Seafan” and “indistinct network” were 22 (43.1%), 8 (15.7%), and 21 (41.2%) at baseline. The median baseline CNV area was 0.361 (0.180–0.897) mm^2^, and the median baseline CNV flow area was 0.299 (0.136–0.646) mm^2^. 16 (31.4%) eyes had lacquer cracks, 45 (88.2%) eyes had posterior staphyloma, 14 (27.5%) eyes had retinoschisis and 17 (33.3%) eyes had DCV. The median hyperreflective area height was 210 (170–286) µm, the median CFT was 300 (219–381) µm and the median SFCT was 54 (26–72) µm.

**Table 1:** Baseline characteristics of eyes with myopic choroidal neovascularization

Parameter	Total eyes (n* =* 51)
Age (years)	53.88 ± 14.01
Gender (n, %)	
Female	38 (74.5%)
Male	13 (25.5%)
Refractive error (D)	− 12.46 ± 3.12
BCVA (logMAR)	0.70 (0.40–1.30)
Anti-VEGF (n, %)	
Ranibizumab	28 (54.9%)
Conbercept	23 (45.1%)
Number of initial injections	2 (1–3)
Number of total injections	3 (2–4)
Follow-up duration (months)	27 (20–41)
CNV location (n, %)	
Subfoveal	33 (64.7%)
Parafoveal	18 (35.3%)
CNV area (mm^2^)	0.361 (0.180–0.897)
CNV flow area (mm^2^)	0.299 (0.136–0.646)
CNV pattern (n, %)	
“Medusa”: “Seafan”: “indistinct network”	22:8:21 (43.1%:15.7%:41.2%)
PSV (n, %)	51 (100.0%)
Lacquer cracks (n, %)	16 (31.4%)
Posterior staphyloma (n, %)	45 (88.2%)
Retinoschisis (n, %)	14 (27.5%)
Dilated choroidal vein (n, %)	17 (33.3%)
Hyperreflective area height (µm)	210 (170–286)
Central foveal thickness (µm)	300 (219–381)
Subfoveal choroidal thickness (µm)	54 (26–72)

*BCVA* = best-corrected visual acuity; *logMAR* = logarithm of the minimum angle of resolution; *anti-VEGF* = anti-vascular endothelial growth factor; *CNV* = choroidal neovascularization; *PS*V = perorating scleral vessels

### Clinical demographic comparisons between mCNV with and without neovascular signal of mCNV around PSV

The presence of PSV was observed in all eyes (51 eyes). Neovascular signal of CNV around PSV was found in 20 eyes (39.2%), and not in 31 eyes (60.8%). A total of three eyes showed differences during observation, and agreement was reached through further observation and discussion. The baseline data and the treatment situation were compared in Table 2.

**Table 2:** Clinical demographic comparisons between mCNVs based on the presence of neovascular signal around PSV

Parameter	Presence of neovascular signal around PSV (n* =* 20)	Absence of neovascular signal around PSV (n* =* 31)	*P* value
Age (years)	51.35 ± 14.35	55.52 ± 13.76	0.304^c^
Gender (n, %)			0.949^a^
Female	15 (75.0%)	23 (74.2%)	
Male	5 (25.0%)	8 (25.8%)	
Refractive error (D)	− 12.41 ± 3.41	− 12.49 ± 2.97	0.928^c^
Baseline BCVA (logMAR)	0.75 (0.43–1.30)	0.52 (0.40–1.00)	0.137^b^
Anti-VEGF (n, %)			0.557^a^
Ranibizumab	12 (60.0%)	16 (51.6%)	
Conbercept	8 (40.0%)	15 (48.4%)	
Number of initial injections	2 (1–3)	2 (1–3)	0.784^b^
Number of total injections	3 (2–4)	2 (2–3)	0.198^b^
Follow- up duration (months)	30.5 (22–45.5)	26 (18–38)	0.284^b^
CNV location (n, %)			0.066^a^
Subfoveal	16 (80.0%)	17 (54.8%)	
Parafoveal	4 (20.0%)	14 (45.2%)	
CNV area (mm^2^)	0.659 (0.178–1.150)	0.310 (0.180–0.670)	0.109^b^
CNV flow area (mm^2^)	0.538 (0.129–0.791)	0.255 (0.136–0.574)	0.177^b^
CNV pattern (n, %)			0.133^a^
“Medusa”: “Seafan”: “Indistinct network”	10:5:5	13:3:16	
	(50.0%:25.0%:25.0%)	(38.7%:9.7%:51.6%)	
Lacquer cracks (n, %)	7 (35.0%)	9 (29.0%)	0.654^a^
Posterior staphyloma (n, %)	17 (85.0%)	28 (90.3%)	0.668^a^
Retinoschisis (n, %)	7 (35.0%)	7 (22.6%)	0.332^a^
Dilated choroidal vein (n, %)	10 (50.0%)	7 (22.6%)	**0.043**^**a**^
Hyperreflective area height (µm)	249.50 (185.25–318.50)	197.00 (150.00–268.00)	0.080^b^
Central foveal thickness (µm)	304.50 (247.25–405.75)	263.00 (206.00–378.00)	0.364^b^
Subfoveal choroidal thickness (µm)	53.00 (24.25–82.25)	54.00 (36.00–68.00)	0.847^b^

*mCNV* = myopic choroidal neovascularization; *PSV* = perorating scleral vessels; *BCVA* = best-corrected visual acuity; *logMAR* = logarithm of the minimum angle of resolution; *anti-VEGF* = anti-vascular endothelial growth factor; *CNV* = choroidal neovascularization

Bold values indicate statistically significant. *P* < 0.05 was considered as significant

^a^Pearson’s χ^2^ test/Fisher's exact test

^b^Mann–Whitney U test

^c^Student’s t-test

The occurrence rate of DCV in the group with neovascular signal around PSV was higher than that in the group without neovascular signal around PSV (50.0% *vs.* 22.6%, *P* = 0.043). The height of hyperreflective area between two groups were not significantly different [249.5 (185.25–318.5) μm *vs.* 197 (150–268) µm, *P* = 0.080)]. There was no significant difference between the other baseline data and the treatment situation.

### Higher rate of recurrence occurred in eyes with neovascular signal around PSV

Twenty of 51 eyes (39.2%) experienced at least one recurrence throughout the follow-up period, of which 10 eyes (50.0%) had a reactivation of original CNV, 4 eyes (20.0%) had further enlargement of the original CNV, and 6 eyes (30.0%) had new CNV formation. For all patients, the recurrence rate within one year was 15.7%. The mean number of anti-VEGF injections was 3.06 times (median, 3.00 times). The overall mean interval between first recurrence was estimated to be 34.43 months (median, 44.00 months, range, 4.00–47.00 months) by Kaplan–Meier survival analysis.

Within one year after treatment, 6 eyes (30.0%) in the group with neovascular signal around PSV had at least one recurrence, and 2 eyes (6.5%) in group without neovascular signal around PSV had at least one recurrence. Higher rate of recurrence occurred in eyes with neovascular signal around PSV (*P* = 0.045) in the first year.

**Fig. 3:**
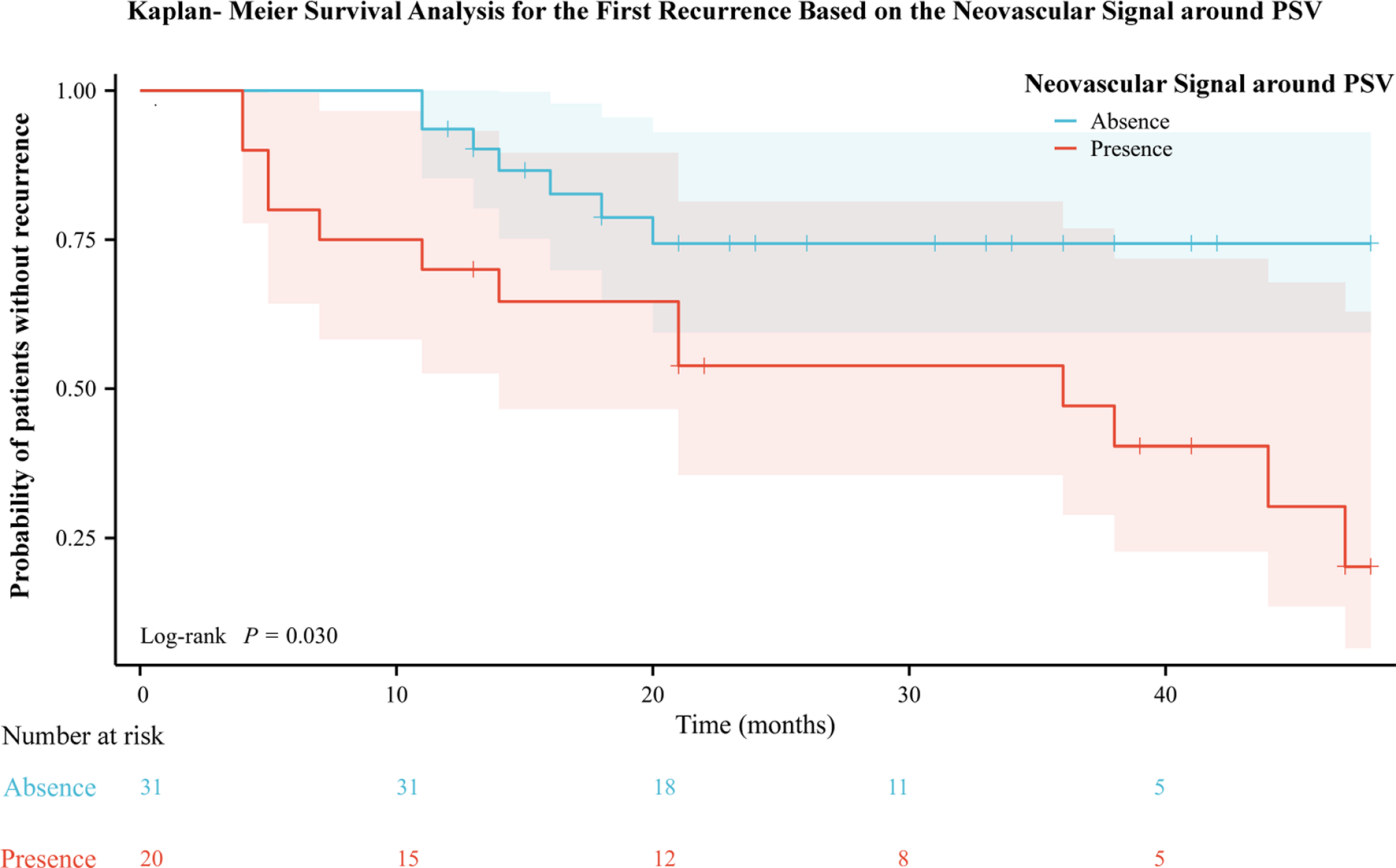
Kaplan–Meier survival curve on recurrence of CNV based on the neovascular signal around PSV during follow-up. The Kaplan–Meier survival curve shows the survival rate each time the event of recurrence in CNV with neovascular signal around PSV group (red line) and CNV without neovascular signal around PSV group (blue line). Log-rank *P* = 0.030. CNV, choroidal neovascularization; PSV, perforating scleral vessel

Kaplan–Meier survival analysis showed that the average interval of first recurrence was 28.68 months (median interval 36.00 months, range, 4.00–47.00 months), and the average interval of first recurrence was 39.55 months (median recurrence interval could not be predicted, range 11.00–20.00 months). The recurrence rate in the group with neovascular signal around PSV was significantly higher than that in the group without neovascular signal around PSV (Fig. 3, Log Rank χ^2^ = 14.712, *P* = 0.030). The one-year recurrence rates of the two groups were 30.0% and 6.5% and the two-year recurrence rates were 46.2% and 25.6%, respectively.

When we conducted survival analysis separately based on drugs, the recurrence probability in the group with neovascular signal around PSV was significantly higher than that in the group without neovascular signal around PSV (Log Rank χ^2^ = 7.332, *P* = 0.007) when treated with conbercept. However, there was no difference in the recurrence probability (Log Rank χ^2^ = 0.364, *P* = 0.547) when treated with ranibizumab.

The risk factors for recurrence were analyzed by Cox proportional hazard regression model (Table 3). Univariate Cox regression analysis was used to analyze the presence or absence of neovascular signal around PSV, baseline BCVA, baseline CNV area, presence or absence of lacquer crack, type of anti-VEGF drugs and SFCT. In univariate analysis, the presence of neovascular signal around PSV and SFCT were associated with the increased risk of CNV recurrence, while baseline BCVA, baseline CNV area, presence or absence of lacquer crack and type of anti-VEGF drugs were not significantly correlated with the risk of CNV recurrence. In the multivariate analysis, the presence of neovascular signal around PSV [*P* = 0.026, HR: 2.904, 95% confidence interval (CI): 1.134–7.436] and SFCT (*P* = 0.034, HR: 0.368, 95% CI: 0.146–0.928) were still significant. The presence of neovascular signal around PSV and SFCT ≤ 50 μm are risk factors for CNV recurrence.

**Table 3:** The risk factors with recurrence of mCNV

Variable	Sub-group	Univariate analysis				Multivariate analysis			
		*P* value	HR	95% CI for HR		*P* value	HR	95% CI for HR	
				5%	95%			5%	95%
Subfoveal choroidal thickness	10–50 μm (47.1%)	**0.0496**	1.000	–	–	**0.034**	1.000	–	–
	51–110 μm (52.9%)		2.526	1.002	6.371		0.368	0.146	0.928
Neovascular signal around PSV	Absence (60.8%)	**0.038**	1.000	–	–	**0.026**	1.000	–	–
	Presence (39.2%)		2.705	1.057	6.922		2.904	1.134	7.436
Anti-VEGF	Conbercept (45.1%)	0.266	1.000	–	–	–	–	–	–
	Ranibizumab (54.9%)		1.658	0.68	4.043	–	–	–	–
Lacquer cracks	Absence (68.6%)	0.116	1.000	–	–	–	–	–	–
	Presence (31.4%)		2.034	0.839	4.930	–	–	–	–
Baseline CNV area	< 0.3 mm^2^ (37.3%)	0.089	1.000	–	–	–	–	–	–
	0.3–0.7 mm^2^ (33.3%)		3.007	1.016	8.899	–	–	–	–
	> 0.7 mm^2^ (29.4%)		1.081	0.328	3.565	–	–	–	–
Baseline BCVA	0.005–0.5 logMAR (31.4%)	0.943	1.000	–	–	–	–	–	–
	0.5–1.0 logMAR (43.1%)		1.191	0.423	3.357	–	–	–	–
	1.0–2.0 logMAR (25.5%)		1.062	0.323	3.485	–	–	–	–

*mCNV* = myopic choroidal neovascularization; *PSV* = perorating scleral vessels; *anti-VEGF* = anti-vascular endothelial growth factor; *CNV* = choroidal neovascularization; *BCVA* = best-corrected visual acuity; *CI* = confidence interval; *HR* = hazard ratio

Bold values indicate statistically significant

*P* < 0.05 was considered as significant

Additional analysis revealed that 15 recurrent eyes with neovascular signal around PSV, 7 (53.8%) eyes were reactivated by the original CNV, 3 (23.1%) eyes had an enlargement of the original CNV, and 3 (23.1%) eyes developed a new CNV that developed away from the original CNV (Table 4). In 7 recurrent eyes without neovascular signal around PSV, 3 (42.9%) eyes were reactivated by the original CNV, 1 (14.3%) eye had an enlargement of the original CNV, and 3 (42.9%) eyes developed a new CNV that developed away from the original CNV. The distribution of recurrence types did not differ (*P* = 0.698). When observing the neovascular signal in CNV which had recurrences, 9 (69.2%) eyes were found PSV around the recurrent CNV in the presence of neovascular signal around PSV group, while 1 (14.3%) eye in the absence of neovascular signal around PSV group was found PSV around the recurrent CNV. However, there was no significant difference between two groups (*P* = 0.057).

**Table 4:** Case series of recurred mCNV patients with neovascular signal around PSV

Patient No	Sex	Age (years)	Affected eye	Baseline BCVA	CNV patterns	Drug	No. of initial injections	First recurrence time (months after diagnosis)/types	PSV around recurrent CNV	BCVA at 12 months
1	F	57	Right	20/100	“Medusa”	Ranibizumab	1	5/Reactivation	Yes	20/67
2	F	57	Left	20/40	“Indistinct network”	Ranibizumab	1	14/Reactivation	Yes	20/67
3	F	85	Left	20/100	“Seafan”	Conbercept	3	4/New CNV	Yes	20/200
4	M	36	Left	20/2000	“Indistinct network”	Conbercept	3	11/Reactivation	Yes	20/50
5	F	64	Right	20/200	“Medusa”	Conbercept	1	5/Reactivation	Yes	20/33
6	F	40	Right	20/40	“Medusa”	Conbercept	5	38/Enlargement	Yes	20/30
7	F	60	Left	20/200	“Medusa”	Conbercept	3	47/Enlargement	Yes	20/100
8	M	48	Left	20/100	“Medusa”	Ranibizumab	2	21/New CNV	No	20/33
9	F	62	Right	20/125	“Medusa”	Conbercept	2	7/Reactivation	Yes	20/40
10	F	71	Right	20/400	“Seafan”	Conbercept	2	36/Reactivation	No	20/50
11	F	54	Left	20/400	“Medusa”	Conbercept	3	21/Enlargement	Yes	20/2000
12	F	53	Right	20/50	“Seafan”	Ranibizumab	3	4/Reactivation	No	20/40
13	F	26	Right	20/40	“Indistinct network”	Ranibizumab	1	44/New CNV	No	20/25

*mCNV* = myopic choroidal neovascularization; *PSV* = perorating scleral vessels; *BCVA* = best-corrected visual acuity; *CNV* = choroidal neovascularization; *F* = female; *M* = male

### Changes of BCVA, hyperreflective area height, CNV area and CNV flow area in eyes with neovascularization around PSV

The changes of BCVA, hyperreflective area height, CNV area and CNV flow area were compared between the two groups through repeated measures analysis of variance (Additional file 1: Table S1). Since the original data is not normally distributed, all the data are converted into normally distributed data by open square root processing.

**Fig. 4:**
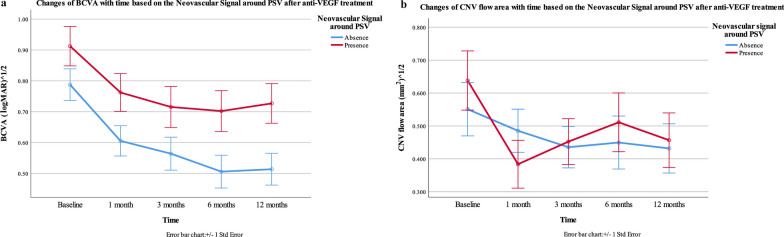
Changes of BCVA and CNV flow area change with time based on the neovascular signal around PSV after anti-VEGF. The x-axis stands for the serial times, y-axis stands for normal distribution data of logMAR BCVA and CNV flow area after converting to the open square root. The influence of groups on CNV flow area was significantly different over time by ANONA (*P*_interaction_ = 0.027). BCVA, best-corrected visual acuity; CNV, choroidal neovascularization; PSV, perforating scleral vessel; anti-VEGF, anti-vascular endothelial growth factor

For BCVA, the main effect of follow-up time was statistically significant (F = 10.507, *P* < 0.001, η^2^_p_ = 0.477), BCVA was significantly improved. The main effect of the group was statistically significant (F = 5.418, *P* = 0.024, η^2^_p_ = 0.100), BCVA of the group with neovascular signal around PSV was worse (Fig. 4a).

For hyperreflective area height, the main effect of follow-up time was statistically significant (F = 12.174, *P* < 0.001, η^2^_p_ = 0.555). The main effect was not statistically significant (F = 0.093, *P* = 0.761, η^2^_p_ = 0.002).

For CNV area, the main effect of follow-up time was statistically significant (F = 4.422, *P* = 0.009, η^2^_p_ = 0.555), and the area of CNV was significantly reduced. The main effect was not statistically significant (*F* = 0.044, *P* = 0.836, η^2^_p_ = 0.002).

For CNV flow area, the main effect of follow-up time was statistically significant (F =  7.711, *P* < 0.001, η^2^_p_ = 0.562). The CNV flow area was significantly reduced. The main effect was not statistically significant (F = 0.029, *P* = 0.866, η^2^_p_ = 0.001). The influence of groups on CNV flow area was significantly different over time (F = 3.326, *P* = 0.027, η^2^_p_ = 0.357) (Fig. 4b). Further simple effect analysis showed that there was no significant difference in simple effect of CNV flow area between the two groups at baseline, 1 month, 3 months, 6 months and 12 months (*P* = 0.479, 0.308, 0.859, 0.613, 0.825, respectively). Multiple comparative findings: in the group with neovascular signal around PSV, the CNV flow area at 1 month, 3 months and 12 months were significantly decreased compared with baseline (*P* < 0.001, *P* = 0.001, *P* = 0.012, respectively), while there was no significant difference at 6 months (*P* = 0.074). In the group without neovascular signal around PSV, the CNV flow area was significantly decreased compared with baseline at three months (*P* = 0.043), while there was no significant difference in the other groups (*P* = 1.000, *P* = 0.163, *P* = 0.134, respectively). In summary, the CNV flow area of the group with neovascular signal around PSV decreased most significantly at one month and then increased, while the CNV flow area of the group without neovascular signal around PSV showed a decreasing trend, but there was no significant difference.

## Discussion

We observed that PSV was mainly detected in patients with mCNV. Not only was the location of PSV discovered around the mCNV, but also the neovascular signal of mCNV was closely related to PSV detected by OCTA. During the follow-up period, 20/51 (39.2%) of the eyes with mCNV recurred. Additionally, when the PSV was around the neovascular signal of CNV, it showed a worse VA result and a greater probability of recurrence with a shorter recurrence time.

Here, we evaluated the neovascularization signals around the PSV for the first time. The neovascular signal of CNV around PSV was found in 20/51 (39.2%) of the eyes. Our study found that the high rate of DCV was detected in the group with neovascular signal around PSV. Xie et al. found the existence of DCV under mCNV and proposed that mCNV might be a vascular unit with input from the short posterior ciliary arteries and drain to the DCV [20]. This may be the reason for the high recurrence rate of mCNV with neovascularization signals around the PSV.

The median follow-up time in this study was 27 (20–41) months. Kaplan–Meier survival analysis estimated that the average interval between initial treatment and first recurrence was 34.43 months (median recurrence time was 44.00 months, range, 4.00–47.00 months). It is suggested that the recurrence rate of mCNV is high after anti-VEGF, and the first recurrence time is about three years. Jain et al. found that 44 (26.4%) eyes recurred after an average follow-up of 16.5 ± 12.86 months [21]. Kang et al. carried out a follow-up for an average of 71.21 ± 26.43 months and found that 35 (46.1%) eyes recurred at least once. The average interval between the initial treatment and the first recurrence was 24.15 ± 18.10 months [5]. Their recurrence time is lower than the results of this study. Considering that their treatment methods include photodynamic therapy (PDT) alone, combinatorial PDT and anti-VEGF treatment, it was found that PDT treatment history is a risk factor for recurrence, and this study only included patients with anti-VEGF alone. We found that the survival curves of the two anti-VEGF drugs were different, and besides the small sample size, other reasons also warrant further investigation.

**Fig. 5:**
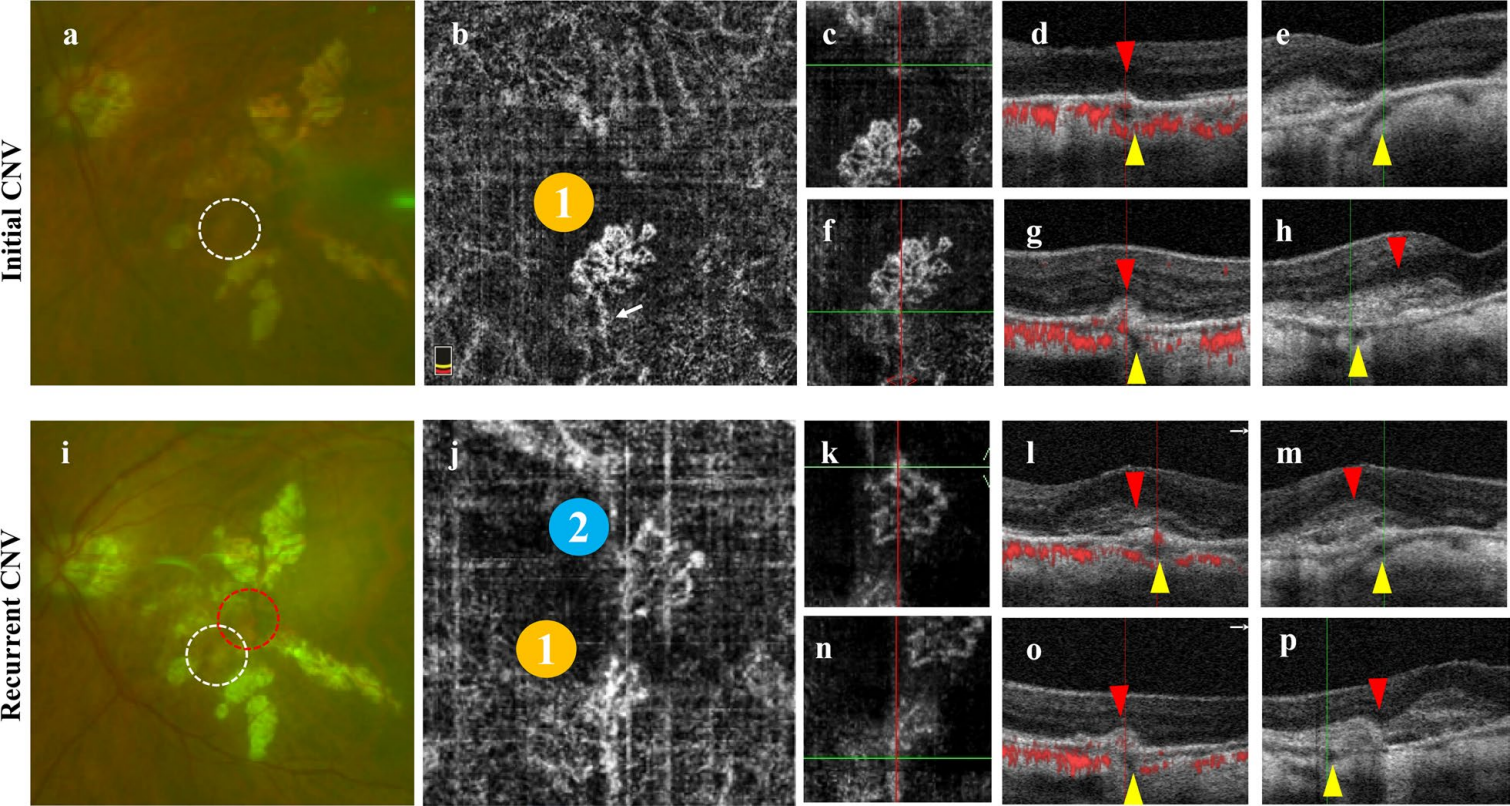
Recurrence of mCNV with neovascular signal around PSV after anti-VEGF therapy. An 83-year-old woman had an active mCNV at initial visit and recurred four months later. The patient underwent one conbercept injection after the onset and another three conbercept injections after the recurrence. Upper row (**a**–**h**) shows the cSLO and OCTA images at initial visit. **a** cSLO shows that initial CNV (white circle) is visualized as a flat, tiny, greyish subretinal lesion. **b** En-face OCTA shows a high-flow network (initial CNV, orange icon 1 including a feeder vessel (white arrow). **c–e** A local magnification of **b**. The cross lines indicate the location of PSV, which is located away from the current CNV. The Bruch’s membrane is intact, however, the RPE layer has a local uplift. **d** is horizontal B-scans and **e** is vertical B-scans. The red arrowhead indicates the uplifted RPE, the yellow arrowhead indicates the PSV. **f–h** Another local magnification of **b**. The cross lines indicate the location of another PSV, corresponding to the feeder vessel of the CNV in **f**. PSV (yellow arrowhead) was identified as a hyporeflective linear region that was found below the CNV (red arrowhead, **g**, **h**) and around a neovascular signal (**g**). Lower row (**i**–**p**) shows the cSLO and OCTA images at the first recurrence visit. **i** A new CNV (red circle) has formed near the original CNV (white circle). **j** En-face OCTA shows the locations of the new CNV (blue icon 2) and the initial CNV (orange icon 1). **k–m** A local magnification of **j**. A new CNV (red arrowhead) has formed. PSV (yellow arrowhead) can be observed below the CNV (red arrowhead), and there is a new neovascular signal around it (**l**). **n–p** The initial CNV partially subsided and the initial neovascular signal cannot be observed (**o**). mCNV, myopic choroidal neovascularization; CNV, choroidal neovascularization; PSV, perforating scleral vessel; anti-VEGF, anti-vascular endothelial growth factor; cSLO, confocal scanning laser ophthalmoscopy; OCTA, optical coherence tomography angiography; RPE, retinal pigment epithelium

When there is neovascular signal of CNV around PSV, the one-year recurrence rate of mCNV is higher and the prognosis is worse. 30.0% (6/20) of the eyes with neovascular signal around PSV had recurrence, which was much higher than that of the group without neovascular signal around PSV (6.5%, *P* = 0.045). Kaplan–Meier survival analysis also showed the same conclusion during the whole follow-up. The recurrence rate of the group with neovascular signal around PSV was significantly higher than that of the group without neovascular signal around PSV (LogRank χ^2^ = 14.712, *P* = 0.030), the mean interval between the first recurrence in the group with neovascular signal around PSV was 28.68 months (median recurrence interval 36.00 months, range, 4.00–47.00 months), and the mean interval between the first recurrence in the group without neovascular signal around PSV was 39.55 months (median recurrence interval could not be predicted, range, 11.00–20.00 months), suggesting that the relationship between PSV and CNV hemodynamics had an impact on the recurrence of mCNV (Fig. 5). Ruiz-Medrano et al. [13] found that compared with mCNV without PSV, mCNV with PSV had a significant increase in the number of relapses after receiving anti-VEGF treatment and received more anti-VEGF intravitreal injections per year. They further analyzed and found that whether PSV was under or in contact with the mCNV had no significant impact on recurrence rate (*P* = 0.60), injection frequency (*P* = 0.52), or annual recurrence frequency (*P* = 0.40). This suggested that the positional relationship between mCNV and PSV is not significantly associated with recurrence. It should be noted that the recurrence interval was defined as at least three months after the original mCNV was stable in our study according to Xie et al. [19] and Jain et al. [21]. Conversely, Ruiz Medrano et al. [22] used an interval of one month for this definition.

**Fig. 6:**
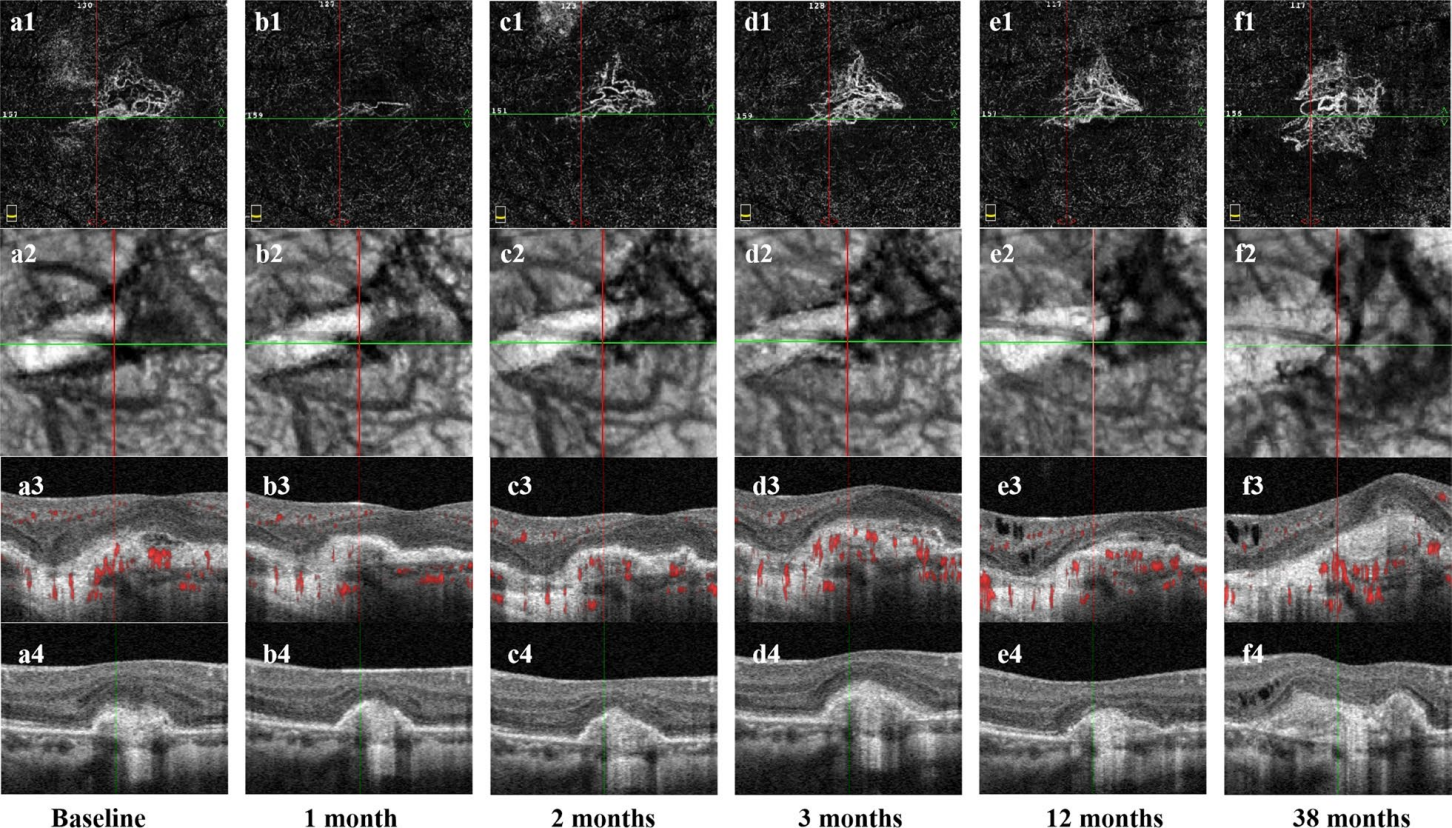
Changes of mCNV with neovascular signal around PSV after anti-VEGF therapy. A 57-year-old woman with an active myopic choroidal neovascularization at initial visit and recurred 38 months later. The patient underwent one ranibizumab injection after the onset, another one ranibizumab injection at three months and another two ranibizumab injections at the first recurrence. Each row represents images of the same follow-up time point. The first line represents en-face OCTA images, the second line represents en-face structure images, the third line represents horizontal cross-sectional OCTA B-scan, and the fourth line represents vertical cross-sectional OCTA B-scan. The baseline CNV (**a1**) shows a “Medusa” pattern, and a PSV (linear low reflection structure, **a3**, **a4**) can be seen under the CNV, corresponding to a black low-reflection lumen-like structure on en-face structure image (**a2**). Neovascular signal (red blood flow signal) can be observed around PSV (**a3**). One month after anti-VEGF therapy, the CNV decreased obviously (**b1**) and the neovascular signal around PSV cannot be observed (**b3**). Two months and three months after anti-VEGF therapy, the CNV area gradually increased (**c1**, **d1**) and the exudation under CNV increased significantly at three months (**d3**), which demonstrated that the mCNV was not stable after therapy. Therefore, she accepted another injection. The CNV was stable at 12 months (**e1**−**e4**). The mCNV recurred at 38 months. The CNV area increased from the baseline (**f1**), the exudation under CNV increased (**f3**, **f4**) and the neovascular signal can be observed around PSV again (**f3**). mCNV, myopic choroidal neovascularization; CNV, choroidal neovascularization; PSV, perforating scleral vessel; anti-VEGF, anti-vascular endothelial growth factor; OCTA, optical coherence tomography angiography

According to the hemodynamic hypothesis, the upregulation of VEGF is the main cause of mCNV, and anti-VEGF therapy is effective in patients with mCNV [23, 24]. BCVA after anti-VEGF treatment was improved regardless of whether there was neovascular signal around PSV, which indicated the effectiveness of anti-VEGF treatment on mCNV and suggested that the upregulation of VEGF was involved in the formation of mCNV. However, when there is neovascular signal around PSV, mCNV is more unstable (Fig. 6). Ishida et al. found that PSV originated from the retrobulbar short posterior ciliary artery through ICGA examination [11]. Louzada et al. found that CNV was connected with PSV through swept-source OCT (SS-OCT) en-face images, suggesting that mCNV may originate from intrascleral blood vessels [25]. The presence of blood flow signals we found at the junction of PSV and CNV further supports this hypothesis, although there is currently no direct histopathological evidence for a relationship. We speculate that when there is a blood flow signal connection between PSV and CNV, this type of mCNV may originate in whole or in part from PSV, and PSV is a blood vessel of arterial origin. In addition to the main factor of elevated VEGF, PSV may also participate in the formation of CNV, which is less sensitive to anti-VEGF treatment. Persistence of PSV flow signal of arterial origin makes CNV less stable and prone to rechallenge.

At the same time, we found that a thinner subfoveal choroid (choroidal thickness ≤ 50 µm) was a risk factor for mCNV recurrence. Similar to our findings, Ahn et al. reported that subfoveal choroid thickness ≤ 47.5 µm was a risk factor for mCNV recurrence [8]. A thinner choroid indicates a higher degree of diffuse chorioretinal atrophy, a more severe degree of macular degeneration [26, 27], and a more severe condition. The loss of large choroidal vessels and/or capillaries in pathological myopia is thought to lead to hypoxia of RPE and glial cells, the latter is an important source of VEGF, and the upregulation of VEGF expression leads to the occurrence of CNV [28]. Some studies have shown that thicker SFCT is an important factor for good visual prognosis with anti-VEGF therapy [9, 29]. Thicker SFCT may have relatively abundant choroidal vessels and more intact choroidal capillaries, resulting in clinically meaningful improvement in visual acuity after treatment, whereas thinner SFCT may lead to choroidal ischemia and subsequent upregulation of angiogenic factors, and thus result in the recurrence of mCNV.

We found that after anti-VEGF treatment, the BCVA of the group with neovascular signal around PSV was worse (F = 9.484, *P* = 0.003, η^2^_p_ = 0.162), suggesting that the blood flow relationship between PSV and CNV has an impact on the development and prognosis of mCNV. At the same time, we observed that in the group with neovascular signal around PSV, the CNV flow area decreased significantly at 1 month, and then increased at 3 months and 6 months, suggesting the instability of CNV with worse prognosis.

The study has several limitations. First, the study is retrospective in nature with a small sample size. To prove the hypothesis, a well-designed prospective study with a large sample size is required. Second, different anti-vascular endothelial growth factor drugs injected into the vitreous cavity may have an effect on the curative effect to some extent. Third, ICGA was not performed in each patient to confirm the source of intrascleral vessels seen on structural OCT images. Further studies with larger samples will be necessary to corroborate our results.

## Conclusions

We found the 39.2% of the eyes had at least one recurrence during the follow-up period. When there is neovascular signal of mCNV around PSV, the results of BCVA are poorer, the recurrence probability is higher, and the recurrence time is shorter, which is one of the risk factors for mCNV recurrence. The results from this study will better guide clinical practice and contribute to a deeper understanding of the pathological mechanisms of mCNV.

### Supplementary Information

**Additional file 1:** **Table S1. **Changes of BCVA, hyperreflective area height, CNV area and CNV flow area during the follow-up period after anti-VEGF treatment.

## Data Availability

The datasets used and analysed during the current study are available from the corresponding author on reasonable request.

## Abbreviations

PSV: Perforating scleral vessel
mCNV: Myopic choroidal neovascularization
CNV: Choroidal neovascularization
anti-VEGF: Anti-vascular endothelial growth factor
BCVA: Best-corrected visual acuity
VA: Visual acuity
OCT: Optical coherence tomography
OCTA: Optical coherence tomography angiography
CFT: Central foveal thickness
SFCT: Subfoveal choroidal thickness
PRN: Pro-re-nata
DCV: Dilated choroidal vein
