# Supplementary material for: Myopic choroidal neovascularization with neovascular signal around perforating scleral vessel prone to recur after anti-VEGF therapy
Source: Eye Vis (Lond). 2024 Feb 7;11:6. doi: 10.1186/s40662-024-00374-5 (PMC10848438; doi:10.1186/s40662-024-00374-5)
Supplement: Supplementary file 1 — Additional file 1: Table S1. Changes of BCVA, hyperreflective area height, CNV area and CNV flow area during the follow-up period after anti-VEGF treatment. [file 40662_2024_374_MOESM1_ESM.docx]

| **Table S1.** Changes of BCVA, hyperreflective area height, CNV area and CNV flow area during the follow-up period after anti-VEGF treatment. | | | | | | | | |  |
| --- | --- | --- | --- | --- | --- | --- | --- | --- | --- |
| Group | BCVA (logMAR) | | | | | F | *P* value | Partial η² | |
|  | Baseline | 1 month | 3 months | 6 months | 12 months |  |  |  | |
| Presence of neovascular signal around PSV | 0.75 (0.43–1.30) | 0.52 (0.30–1.00) | 0.46 (0.22–1.00) | 0.52 (0.15–0.96) | 0.46 (0.22–1.00) | - | - | - | |
| Absence of neovascular signal around PSV | 0.52 (0.40–1.00) | 0.30 (0.15–0.70) | 0.30 (0.15–0.52) | 0.22 (0.15–0.40) | 0.22 (0.10–0.40) | - | - | - | |
| Main effect of PSV | - | - | - | - | - | 5.418 | 0.024 | 0.100 | |
| Main effect of follow-up time | - | - | - | - | - | 10.507 | <0.001 | 0.477 | |
| Interaction of PSV and follow-up time | - | - | - | - | - | 0.888 | 0.479 | 0.072 | |
| Group | Hyperreflective area height (µm) | | | | | F | *P* value | Partial η² | |
|  | Baseline | 1 month | 3 months | 6 months | 12 months |  |  |  | |
| Presence of neovascular signal around PSV | 210 (174–300) | 136 (98–170) | 151 (81–190) | 147 (68–190) | 127 (51–190) | - | - | - | |
| Absence of neovascular signal around PSV | 197 (139–275.5) | 112 (77–192.5) | 118 (74–174) | 104 (71–185) | 104 (61.5–181) | - | - | - | |
| Main effect of PSV | - | - | - | - | - | 0.0093 | 0.761 | 0.002 | |
| Main effect of follow-up time | - | - | - | - | - | 12.174 | <0.001 | 0.555 | |
| Interaction of PSV and follow-up time | - | - | - | - | - | 0.775 | 0.548 | 0.074 | |
| Group | CNV area (mm^2^) | | | | | F | *P* value | Partial η² | |
|  | Baseline | 1 month | 3 months | 6 months | 12 months |  |  |  | |
| Presence of neovascular signal around PSV | 0.364 (0.115–1.214) | 0.220 (0.031–0.616) | 0.337 (0.061–0.681) | 0.337 (0.059–0.811) | 0.276 (0.074–0.757) | - | - | - | |
| Absence of neovascular signal around PSV | 0.306 (0.144–0.632) | 0.223 (0.105–0.672) | 0.233 (0.108–0.593) | 0.226 (0.118–0.588) | 0.274 (0.065–0.582) | - | - | - | |
| Main effect of PSV | - | - | - | - | - | 0.044 | 0.836 | 0.002 | |
| Main effect of follow-up time | - | - | - | - | - | 4.422 | 0.009 | 0.555 | |
| Interaction of PSV and follow-up time | - | - | - | - | - | 2.727 | 0.055 | 0.331 | |
| Group | CNV flow area (mm^2^) | | | | | F | P value | Partial η² | |
|  | Baseline | 1 month | 3 months | 6 months | 12 months |  |  |  | |
| Presence of neovascular signal around PSV | 0.299 (0.082–0.858) | 0.164 (0.021–0.469) | 0.241 (0.044–0.459) | 0.241 (0.045–0.646) | 0.156 (0.047–0.573) | - | - | - | |
| Absence of neovascular signal around PSV | 0.251 (0.111–0.557) | 0.173 (0.081–0.556) | 0.199 (0.071–0.446) | 0.192 (0.096–0.489) | 0.220 (0.047–0.451) | - | - | - | |
| Main effect of PSV | - | - | - | - | - | 0.029 | 0.886 | 0.001 | |
| Main effect of follow-up time | - | - | - | - | - | 7.711 | <0.001 | 0.562 | |
| Interaction of PSV and follow-up time | - | - | - | - | - | 3.326 | 0.027 | 0.357 | |
| BCVA = best-corrected vision acuity; logMAR = logarithm of the minimum angle of resolution; CNV = choroid neovascularization; anti-VEGF = anti-vascular endothelial growth factor; PSV = perforating scleral vessel; *P*<0.05 was considered as significant by repeated measures ANONA. LogMAR BCVA, HRF height, CNV area and CNV flow area were processed by open square root and converted into normal data for analysis. | | | | | | | | | |
